# Recurrence associated with 2-year visual acuity after subretinal tissue plasminogen activator for submacular hemorrhage in neovascular age-related macular degeneration

**DOI:** 10.1007/s10384-025-01276-2

**Published:** 2025-09-08

**Authors:** Mie Tanaka, Manabu Miyata, Masayuki Hata, Sotaro Ooto, Hiroshi Tamura, Ai Kido, Naoko Ueda-Arakawa, Masahiro Miyake, Ayako Takahashi, Yuki Muraoka, Akitaka Tsujikawa

**Affiliations:** Department of Ophthalmology and Visual Sciences, Kyoto University Graduate School of Medicine, 54 Shogoin Kawahara-cho, Sakyo-ku, Kyoto City, Kyoto Prefecture 606-8507 Japan

**Keywords:** Neovascular age-related macular degeneration, Photodynamic therapy, Recurrence, Submacular hemorrhage, Tissue plasminogen activator

## Abstract

**Purpose:**

To identify predictors of the 2-year best-corrected visual acuity (BCVA) after subretinal tissue plasminogen activator (tPA) injection for massive submacular hemorrhage (SMH) complicating neovascular age-related macular degeneration (nAMD).

**Study design:**

A prospective, observational study.

**Methods:**

This study included consecutive eyes with massive SMH and nAMD that underwent vitrectomy with subretinal tPA injection and follow-up for 2 years. We analyzed the correlation between the 2-year BCVA and other parameters, including baseline BCVA, SMH height, SMH size, and SMH recurrence.

**Results:**

This study analyzed 20 eyes of 20 patients (72.5 ± 7.2 years). Two years after surgery, the mean logarithm of the minimum angle of resolution (logMAR) BCVA changed from 0.72 (Snellen equivalent, 20/105) ± 0.40 at baseline to 0.80 (Snellen equivalent, 20/126) ± 0.92. The BCVA did not change significantly during the 2-year observation period (*P* = 0.39). Compared to baseline, the 2-year BCVA improved in 11 eyes (55%) and declined in 6 eyes (30%) by more than 0.30 logMAR, including all five eyes with recurrence. The 2-year BCVA was correlated only with recurrence (*P* < 0.001, β = 0.85).

**Conclusions:**

This study suggests that recurrence was a robust determinant of poor 2-year BCVA after vitrectomy with subretinal tPA injection for SMH complicating nAMD and that subretinal tPA injection was effective in most cases, without recurrence. Our findings highlight the importance of establishing methods for preventing and controlling recurrence to maintain long-term BCVA.

## Introduction

Submacular hemorrhage (SMH) sometimes occurs as a complication in eyes with neovascular age-related macular degeneration (nAMD), accompanied by the sudden loss of central vision. SMH reportedly occurred in 30% of eyes with a subtype of nAMD known as polypoidal choroidal vasculopathy (PCV) during a 10-year follow-up period, despite being under control by anti-vascular endothelial growth factor (VEGF) therapy and/or photodynamic therapy (PDT) [1]. The natural course of SMH is expected to be extremely severe, as evidenced by the drastic decline in the mean best-corrected visual acuity (BCVA) from 20/200 to 20/2000 without treatment in a 2-year observational study [2].

Since the toxicity of iron (present in hemoglobin) to the photoreceptor is considered to be one of the causes of poor visual prognosis [3], early displacement of the SMH from the macula using vitrectomy with subretinal tissue plasminogen activator (tPA) injection has been performed for its management [4–8]. The mean 1-year BCVA (20/78) appeared to improve relative to baseline (20/115) after subretinal tPA injection [8]; however, to our best knowledge, observational studies with a follow-up period of 2 years or more have not been reported. Furthermore, the previous study found that the recurrence of SMH was a strong factor associated with the 1-year BCVA [8].

This study builds upon a previous 1-year study [8], aiming to demonstrate the 2-year visual outcomes after vitrectomy with subretinal tPA injection for SMH complicating nAMD and to identify the factors associated with the 2-year BCVA by extending the observation period for a subset of cases.

## Materials and methods

The ethics committee of the Kyoto University Graduate School of Medicine (Kyoto, Japan) approved this prospective, observational study (approval number, R0532). All study protocols adhered to the tenets of the Declaration of Helsinki, and written informed consent was obtained from all participants.

### Participants

This study enrolled consecutive eyes with massive SMH involving the fovea occurring as a complication of nAMD, which were treated using vitrectomy with subretinal tPA injection performed by the same surgeon (Manabu Miyata) at Kyoto University Hospital between June 2018 and July 2022. The necessity of surgery was judged by one retinal specialist (Manabu Miyata) based on the SMH area, SMH height, and SMH status, including reflective intensity obtained using optical coherence tomography (OCT). The exclusion criteria were dropout from follow-up for 2 years, lack of adequate examination before surgery due to preoperative dense vitreous hemorrhage or severe dementia, and BCVA ≤ light perception. When both eyes of the same patient met the criteria, the eye that was initially treated was selected for analysis.

Before surgery, we performed comprehensive ophthalmological examination, including BCVA assessment using the Landolt chart, intraocular pressure measurement, axial length measurement using partial coherence interferometry (IOLMaster; Carl Zeiss Meditec,), slit-lamp biomicroscopy with a fundus diagnosis lens, color fundus photography (TRC-NW8F; Topcon; and/or Optos), and OCT (Spectralis; Heidelberg Engineering). At each follow-up examination, we performed BCVA assessment, intraocular pressure measurement, slit-lamp microscopy, color fundus photography, and OCT. We analyzed the BCVA measured at baseline and 2 weeks, 3 months, 1 year, and 2 years after surgery.

At baseline, SMH height was measured as the distance between the outer border of the sensory retina and the surface of the retinal pigment epithelium at the fovea on OCT images, while SMH size (disc diameter) was assessed using fundus color photographs by one investigator (MT). After surgery, data on the recurrence of massive SMH involving the fovea, number of anti-VEGF therapies, and number of PDT sessions were assessed based on the medical records. Disease duration was calculated as the time interval between onset (based on patient-reported episodes) and surgery.

Surgery

We performed surgery using previously reported methods [8]. Briefly, surgery was implemented as early as possible after the patient’s initial visit without discontinuation of antiplatelet or anticoagulant drug intake. When cataract was present, we first performed cataract surgery using the phacoemulsification technique and intraocular lens implantation under local anesthesia using instillation of 4% lidocaine eye drops and sub-Tenon’s capsule injection of 2% lidocaine. We performed 27-gauge pars plana vitrectomy using the Constellation Vision System (Alcon Laboratories, followed by subretinal injection of 0.1–0.3 mL (4,000–12,000 IU) monteplase (Cleactor; Eisai Co) administered through a 38-gauge cannula (PolyTip Cannula 27g/38g; MedOne) and 1-mL silicon oil syringe (MicroDoseTM Injector; MedOne) connected to Constellation. Finally, we performed fluid/air exchange of approximately 50% of the vitreous cavity, using 25% SF_6_ gas for air exchange. After consecutive surgeries, we instructed the patients to maintain a prone position for a few days.

### Post-surgical therapy

We administered a single dose of intravitreal anti-VEGF therapy, using aflibercept 2 mg (Eylea; Bayer) within 1 week of surgery for all eyes. After the initial injection, anti-VEGF therapy was continued or PDT was performed per the surgeon’s discretion based on each patient’s status and demand. We adhered to the Japanese Age-Related Macular Degeneration Trial’s recommendation that PDT be used for eyes with a baseline BCVA ranging from 20/40 to 20/200 [9]; however, the Ophthalmic PDT Study Group in Japan also deemed PDT to be acceptable for cases with good BCVA (> 20/40) and potential risks [10]. Since a previous study reported that PDT followed by anti-VEGF therapy lowered the risk of massive SMH in PCV [1], we performed PDT with anti-VEGF therapy (Ranibizumab BS, Senju Pharmaceuticals) for eyes with PCV, particularly those with BCVA ≤ 20/40, in patients who provided informed consent. However, because anti-VEGF therapy has a higher effect on the long-term maintenance of BCVA in eyes with the non-pachychoroid phenotype compared with PDT [11], we primarily selected anti-VEGF therapy for eyes with this phenotype. We mainly used a pro re nata regimen for anti-VEGF monotherapy. If a massive SMH recurred, our treatment strategy entailed surgery for eyes with BCVA reduction or anti-VEGF therapy for eyes without BCVA reduction, after consulting with the patient, because a multicenter study suggested the efficacy of tPA for eyes with BCVA reduction and that of anti-VEGF therapy for eyes without BCVA reduction [12].

### Statistical analysis

We presented data as the mean ± standard deviation, where applicable. We converted the BCVA into the logarithm of the minimum angle of resolution (logMAR) values for statistical analysis. As reported previously [13], logMAR values of 2.6, 2.7, 2.8, and 2.9 were assigned to the BCVA categories of counting fingers, hand motion, light perception, and no light perception, respectively. We analyzed the changes in logMAR BCVA at baseline and 2 weeks, 3 months, 1 year, and 2 years after surgery using analysis of variance with repeated measures; if significant, we subsequently performed post hoc analysis with Bonferroni correction. We established a cut-off value of 0.30 logMAR for BCVA change, corresponding to 15 letters on the ETDRS chart, as reported previously [14, 15]. We performed univariable correlation analyses between the 2-year logMAR BCVA and other parameters using Spearman’s correlation coefficient; furthermore, we performed multivariable correlation analyses (stepwise linear regression) using the 2-year logMAR BCVA as the dependent variable. We used SPSS (version 27.0; IBM) for all statistical analyses. *P*-values < 0.05 denoted statistical significance.

## Results

**Figure 1.**
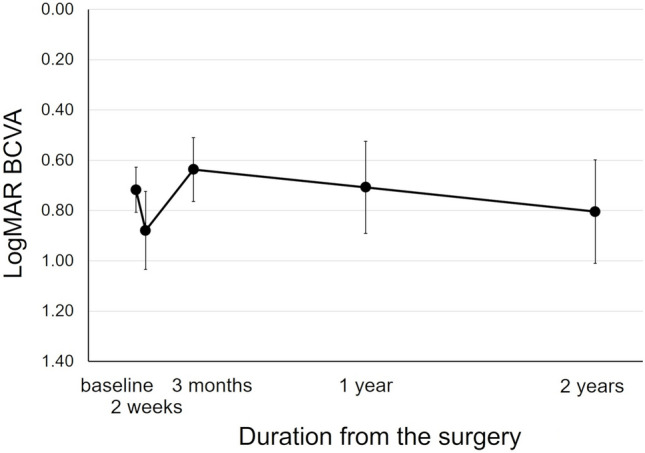
Change in the logarithm of the minimum angle of resolution (logMAR) best-corrected visual acuity (BCVA). The logMAR BCVA did not change significantly during the 2-year observation period (P = 0.39). The bars represent standard errors.

Twenty eyes of 20 patients were analyzed in this study (Table 1), after excluding 17 patients who dropped out, two with severe dementia, and one whose BCVA was no light perception. Seventeen eyes in this study overlapped with the sample of the previous 1-year study [8]. The patients’ mean age was 72.5 ± 7.2 (range, 62–91) years. The mean disease duration between onset and surgery was 7.0 ± 4.5 (range, 2–15) days. The mean logMAR BCVA changed from 0.72 (Snellen equivalent, 20/105) ± 0.40 at baseline to 0.80 (Snellen equivalent, 20/126) ± 0.92 2 years after surgery. The BCVA did not change significantly during the 2-year observation period (*P* = 0.39; Fig. 1). Compared to baseline, the 2-year BCVA improved in 11 eyes (55%) by more than 0.30 logMAR and declined in six eyes (30%) by more than 0.30 logMAR, including all five eyes with recurrence, and remained stable within 0.30 logMAR change in three eyes (15%).

**Table 1.** Participants’ characteristics

Eyes, n (patient, n)	20 (20)
Age at surgery, years (range)	72.5 ± 7.2 (62–91)
Male sex, n	9 (45%)
Axial length, mm (range)	24.17 ± 1.88 (20.97–28.72)
Disease duration (onset–surgery), days (range)	7.0 ± 4.5 (2–15)
LogMAR BCVA (Snellen)	
Baseline	0.72 (20/105) ± 0.40
2-week	0.88 (20/152) ± 0.69
3-month	0.64 (20/87) ± 0.57
1-year	0.71 (20/103) ± 0.82
2-year	0.80 (20/126) ± 0.92
SMH height at baseline, μm	384.4 ± 226.2
SMH size at baseline, disc diameters	4.3 ± 2.1
SMH recurrence during 2 years, n	5 (25%)
Duration between surgery and SMH recurrence (n = 5), days (range)	161 ± 132 (55–392)
Number of anti-VEGF therapies after surgery (range)	7.0 ± 1.7 (2–12)
PDT after surgery, n	
Recurrence (−) (n = 15)	5 (33%)
Recurrence (+) (n = 5)	
Before recurrence	3 (60%)
After recurrence	2 (40%)

Data are presented as means ± standard deviations where applicable.

*logMAR BCVA* logarithm of the minimal angle of resolution best-corrected visual acuity, *SMH* submacular hemorrhage, *PDT* photodynamic therapy

At baseline, the mean SMH height was 384.4 ± 226.2 μm, and the mean SMH size was 4.3 ± 2.1 disc diameters. Massive SMH recurred in five patients; the mean duration between surgery and recurrence was 161 ± 132 (range, 55–392) days during the 2-year postoperative period.

### Post-surgical therapy

**Figure 2.**
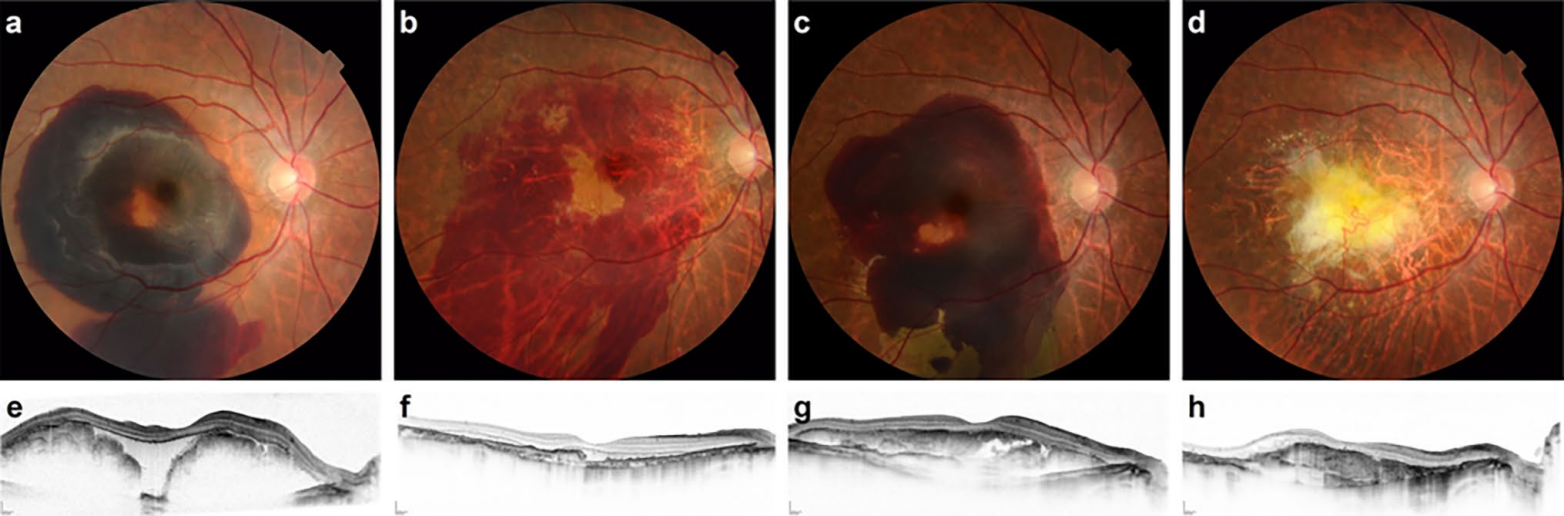
Representative images of an eye with recurrence treated without additional subretinal tissue plasminogen activator injection. Color fundus photographs (**a–d**) and optical coherence tomography (OCT) images (**e–h**) of the right eye of a man in his 70s. **a, e** Preoperative images. The best-corrected visual acuity (BCVA) was 20/67. **a** Submacular hemorrhage (SMH) measuring 4 disc diameters in size and involving the fovea can be observed. (**e**) The height of the SMH is 1090 μm. **b, f** Images acquired 2 weeks after surgery. The BCVA was 20/67. **b** Displacement of the massive SMH can be observed. (**f**) A thin and faint residual SMH is evident. **c, g** Images showing recurrence 3 months after surgery. The BCVA was 20/67. Three monthly intravitreal injections of aflibercept 2 mg were administered before recurrence. Since the BCVA did not change before and after recurrence, we decided to deliver additional injections after discussion with the patient. **c** SMH recurrence can be observed. **g** The SMH height is much lower than that at baseline. **d, h** Images acquired 2 years after surgery. Additional one intravitreal aflibercept 2 mg and five intravitreal brolucizumab 6 mg injections were administered between recurrence and this juncture. The BCVA worsened to 20/333. **d** SMH is absent, but fibrosis is observed. **e** Subretinal fibrosis can be observed.

After surgery, all eyes received anti-VEGF therapy (mean number of injections, 7.0 ± 1.7; range, 2–12), and 10 eyes underwent PDT (1 session for all eyes). Five (33%) of 15 eyes without recurrence underwent PDT. Among the five eyes with recurrence, three eyes (3/5; 60%) underwent PDT before recurrence, and all three eyes underwent PDT within 1 month after surgery. In these three eyes, recurrence occurred 3, 5, and 12 months after PDT, respectively. Recurrence occurred 1 month after intravitreal 2mg aflibercept injection in the other two eyes that did not receive PDT before recurrence. In all five eyes, SMH recurred even though the absence of exudates attributable to PDT and/or anti-VEGF therapy was confirmed at the latest visit. Among the five eyes with recurrence, two eyes (2/5; 40%) underwent PDT 2 months and 17 months after recurrence, respectively. After recurrence, one eye received vitrectomy with subretinal tPA injection again; however, the other four patients did not agree to reoperation because their BCVA remained nearly unchanged before and after recurrence (Fig. 2).

### Correlation analyses

Spearman’s correlation coefficient analysis revealed that the 2-year logMAR BCVA was correlated only with SMH recurrence (*P* < 0.001, r = 0.71). In contrast to a previous study on the 1-year outcomes [8], the 2-year logMAR BCVA was not significantly correlated with age (*P* = 0.099, r = 0.38) or baseline logMAR BCVA (*P* = 0.56, r = 0.14). Multivariable analysis also reiterated the correlation between the 2-year logMAR BCVA and recurrence (*P* < 0.001, β = 0.85; Table 2).

**Table 2.** Correlation between the 2-year logMAR BCVA and the studied parameters

	Univariable Analysis		Multivariable Analysis	
	***P***	β	*P*	β
Age	0.095	0.38	-	-
Sex (1, men; 2, women)	0.90	0.03	-	-
Axial length	0.40	−0.20	-	-
Disease duration (onset–surgery period)	0.55	−0.14	-	-
LogMAR BCVA at baseline	0.46	0.18	-	-
SMH height at baseline	0.76	−0.07	-	-
SMH size at baseline	0.07	0.41	-	-
Cataract surgery with vitrectomy (0, −; 1, +)	0.75	0.07	-	-
Number of postoperative anti-VEGF therapies	0.65	0.11	-	-
Number of postoperative PDT sessions	0.08	0.41	-	-
SMH recurrence (0, −; 1, +)	< 0.001*	0.85	< 0.001*	0.85

*logMAR BCVA* logarithm of the minimal angle of resolution best-corrected visual acuity, *SMH* submacular hemorrhage, *PDT* photodynamic therapy

^*^Statistically significant (*P* < 0.05)

## Discussion

The present study demonstrated that the BCVA did not significantly change during the 2 years after vitrectomy with subretinal tPA injection for eyes with nAMD complicated by SMH. This finding implies the effectiveness of a series of therapies, including subretinal tPA injection, in contrast to a previous study that observed the natural course of SMH in which the mean BCVA plummeted drastically from 20/200 to 20/2000 over 2 years [2]. Furthermore, recurrence was the most important factor correlated with the 2-year BCVA (*P* < 0.001, β = 0.85), and the importance of recurrence was stronger than reported by a previous 1-year observational study (recurrence, *P* < 0.001, β = 0.65; age, *P* = 0.007, β = 0.39) [8]. Further studies are needed to establish methods to prevent SMH recurrence and ensure the long-term maintenance of BCVA.

Controlling SMH recurrence is also key, as gleaned from our findings; however, no evidence has been established. A previous study shows that PDT followed by anti-VEGF therapy lowered the risk of massive SMH (HR = 0.242; *P* = 0.047) in eyes with PCV [1]. Although we selected PDT or anti-VEGF therapy based on the patient’s status and wishes, 33% of eyes without recurrence and 60% of eyes with recurrence underwent PDT (the latter, before recurrence), which were similar in varying durations. Therefore, our results do not suggest that PDT was effective in preventing recurrence. Further prospective randomized studies on the effectiveness of PDT are warranted. Furthermore, we complied with the wishes of four of the five patients with recurrence who did not wish to undergo reoperation with subretinal tPA injection because of almost no decline in their BCVA before and after recurrence; therefore, we did not perform surgery again. However, considering the results of our 2-year observational study, surgery may be recommended even in eyes without BCVA decline at the time of recurrence.

In this 2-year observational study of eyes with SMH complicating nAMD treated with post-surgical initial therapy of intravitreal injection of aflibercept 2 mg, the BCVA did not change significantly, whereas previous studies report that the BCVA improved significantly 2 years after initial treatment with intravitreal injection of aflibercept 2 mg in eyes with nAMD [15–17]. Based on observation of the mean BCVA, SMH secondary to nAMD appeared to culminate in poor long-term visual outcomes. However, in 55% of eyes, the BCVA improved by more than 0.30 logMAR (corresponding to 15 letters), whereas in 30% (six) of eyes, including all five eyes with recurrence, the BCVA declined by more than 0.30 logMAR. If SMH recurrence could be prevented, the disease course would improve after vitrectomy with subretinal tPA injection.

The question of which treatments were effective for preventing SMH recurrence as the post-surgical therapy remains. We administered an intravitreal injection of aflibercept 2 mg as the initial treatment after surgery. Brolucizumab 6 mg, faricimab 6 mg, and aflibercept 8 mg have recently become available. Although the long-term safety of aflibercept 2 mg has been demonstrated [15], a short-term study suggests that both the effectiveness of brolucizumab and of faricimab was higher than that of aflibercept 2 mg [18]. Since the clearance of anti-VEGF increases in vitrectomized eyes, longer-acting agents may be desired [19]. A recent study reports that aflibercept 8 mg could extend dosing intervals compared with aflibercept 2 mg [20]. New agents may be more effective in preventing recurrence. Furthermore, cluster-type PCV, characterized by multiple aggregative polypoidal lesions, is at a high risk for massive SMH [1]. Therefore, PDT may be better suited for the regression of polypoidal lesions in PCV [21]. Further prospective studies are needed to investigate treatments and regimens that could serve as effective post-surgical therapy.

In this study, massive SMH recurred 161 ± 132 (55–392) days after the initial surgery, which was unexpectedly long. The elimination half-life of monteplase (Cleactor), used as tPA in the present study, is 23.66 min; therefore, the drug should not affect recurrence. Despite anti-VEGF therapy and/or PDT, which would have stabilized macular neovascularization activity, SMH recurred, as reported previously [1]. Even after SMH is displaced and disappears from the fovea, and macular neovascularization activity becomes apparently stable, long-term follow-up is necessary.

Initial surgery was performed 7.0 ± 4.5 (2–15) days after onset. Premature surgery using tPA might result in severe hemorrhage, whereas delayed surgery might elevate the risk of irreversible photoreceptor damage due to iron toxicity; therefore, the timing of subretinal tPA injection is difficult to judge. A previous study suggests that the best time for surgery is 7–10 days after onset [22]. Another recent study reports that vitrectomy with subretinal balanced saline and air injection without tPA was effective within 1 week of symptom manifestation in most cases [3.60 ± 2.78 (1–12) days] [23]. Subretinal tPA injection might not be needed within a week of onset. A study comparing vitrectomy with subretinal balanced saline injection and vitrectomy with subretinal tPA injection for eyes with SMH within 1 week is needed.

Although the point estimates cannot be directly compared in a statistically rigorous way, the relationship between final BCVA and SMH recurrence seemed more pronounced in the second year than in the first year previously reported (β = 0.85 and 0.65, respectively) [8]. We also observed a poorer 1-year visual outcome in the current study (baseline logMAR, 0.72 ± 0.40; 1-year logMAR, 0.71 ± 0.82) compared to the improvement seen in the 1-year previous study (baseline logMAR, 0.82 ± 0.40; 1-year logMAR, 0.59 ± 0.65) [8]. These discrepancies likely stem from differences in the patient cohorts. The 1-year study included cases of both nAMD and retinal arterial macroaneurysm (RAM), whereas the current 2-year study focused exclusively on nAMD. RAM is characterized by a lower recurrence rate and favorable visual outcomes, largely due to effective intraoperative photocoagulation; a meta-analysis reported a RAM closure rate of 96% [24]. In contrast, macular neovascularization in nAMD is not definitively cured, despite various treatments. Therefore, for the long-term maintenance of BCVA, particularly in nAMD, SMH recurrence prevention is paramount.

This study has some limitations. First, the sample size was small, necessitating future studies with larger sample sizes. Second, optimal post-surgical therapies have not been established. We acknowledged and respected the patients’ intentions and feelings after informing them of the disease status and selected therapies based on our treatment policy, as mentioned above. Third, 17 eyes in this study overlapped with the sample of our previous 1-year observational study because this was a continuation study [8], which might have affected the results. However, the longer observation period enabled better clarification that the prevention of recurrence is key to maintaining long-term BCVA in SMH. Fourth, we could not accurately classify the subtype of nAMD because most cases of SMH treated at our hospital were referred for emergency surgery from other hospitals. Further studies with a large sample size that focus only on cases in which subtypes have already been classified are needed.

In conclusion, this 2-year observational study suggests that recurrence was a robust determinant of poor BCVA after vitrectomy with subretinal tPA injection for SMH complicating nAMD. Furthermore, subretinal tPA injection was effective in most cases unless there was a recurrence. Our findings highlight the importance of establishing methods for preventing and controlling recurrence to maintain BCVA in the long term.
